# Blended-ALMAMAR app for inpatient mental health care for refugees: study protocol for a multicenter implementation study within the I-REACH consortium (Internet based REfugee mentAl healtH Care)

**DOI:** 10.1186/s12913-023-10403-z

**Published:** 2023-12-13

**Authors:** Isabelle Reinhardt, Laura Schmidt, Dirk Reske, Jürgen Zielasek, Gracia Braun, Maria Böttche, Johanna Boettcher, Sebastian Burchert, Heide Glaesmer, Christine Knaevelsrud, Alexander Konnopka, Louisa Muntendorf, Laura Nohr, Sophia Paskuy, Babette Renneberg, Susan Sierau, Nadine Stammel, Birgit Wagner, Tina Wirz, Euphrosyne Gouzoulis-Mayfrank

**Affiliations:** 1 LVR-Institute for Research and Education - Section Healthcare Research, Cologne, Germany; 2 Medical Faculty, Heinrich Heine University Düsseldorf, Düsseldorf, Germany; 3 Division of Clinical Psychological Intervention, Freie Universitaet Berlin, Berlin, Germany; 4 Clinical Psychology and Psychotherapy, Freie Universitaet Berlin, Berlin, Germany; 5 Clinical Psychology and Psychotherapy, Psychologische Hochschule Berlin, Berlin, Germany; 6 Medical Psychology and Medical Sociology, University of Leipzig, Leipzig, Germany; 7 Health Economics and Health Services Research, University Medical Center Hamburg-Eppendorf, Hamburg, Germany; 8 Medical School Berlin, Clinical Psychology and Psychotherapy, Berlin, Germany

**Keywords:** Implementation, Internet-based intervention, Blended care, Inpatient mental healthcare, Refugees

## Abstract

**Background:**

Refugees are at high risk for developing mental illnesses. Due to language and cultural barriers, there is need for specifically adapted therapeutic procedures for refugees in inpatient mental health care settings. Internet-based applications in refugee mother tongues have the potential to improve the outcomes of mental health care for this vulnerable population. The key research question of the present implementation study is whether the newly developed “blended ALMAMAR” app for Arabic and Farsi speaking refugees in Germany is used and accepted by patients and professionals in routine inpatient mental health care (blended care).

**Methods:**

We present the design of an observational, prospective multicenter implementation study in eight psychiatric hospitals. We plan to recruit 100 Farsi or Arabic speaking refugees receiving in-patient treatment due to depression, anxiety disorder, posttraumatic stress disorder or substance use disorders. These patients will get access to the “blended ALMAMAR” app during their inpatient stay in a blended-care approach. We will assess the usage (e.g., duration and frequency of use of the app) as well as subjective acceptability and usability of the intervention. To identify sociodemographic and clinical factors associated with “blended ALMAMAR” usage, we will also perform clinical and questionnaire assessments.

**Discussion:**

The newly developed “blended ALMAMAR” app may help to close communication gaps for the hard-to reach and vulnerable group of refugees in inpatient mental health care. It is the first blended-care intervention that addresses severely mentally ill refugees in an inpatient psychiatric setting in Germany.

**Trial registration:**

The trial was registered in the German Clinical Trials Register on November 11, 2021 (DRKS00025972) and adapted on November 14, 2023.

## Background

The number of refugees and asylum applicants in Europe and Germany remains high (approximately 1 million refugees from the Ukraine and more than 217.000 first applications for asylum in 2022 in Germany). People from Arabic- and Farsi-speaking countries (e.g., Syria, Afghanistan, Iraq, Iran) are among those who submitted the highest number of asylum applications in Germany [1]. Refugees are at a high risk for developing mental illnesses due to the burden of pre-, peri- and postmigration stressors [2, 3]. The prevalence of posttraumatic stress disorder (PTSD) among refugees is approximately 30% and there is an equally high prevalence of depression [4–7]. Accordingly, the high number of refugees from different countries is a challenge for European mental health care systems [e.g. 2] and there is a need to implement effective mental healthcare approaches [8]. Treatment methods and support services need to be adapted to the specific needs of refugee populations [9]. Internet-based applications have the potential to improve the outcomes of mental healthcare for refugees by providing culturally sensitive, cost-effective, native-language services [9].

Numerous studies have demonstrated beneficial effects of stand-alone internet-based interventions *and* blended online interventions in combination with face-to-face psychotherapy in non-refugee populations. A systematic review of 44 blended care studies for outpatient treatment for mental disorders in adult populations suggested that blended care approaches were clinically effective [10]. To date, three studies implemented internet-based applications in psychiatric inpatient settings in Germany and reported promising results for (non-refugee) patients with depression [11–13].

Refugees now represent a large group in inpatient mental healthcare [14]. However, language and cultural factors are major barriers to the delivery and uptake of mental health services: participation in group therapies is difficult, specific therapeutic programs are lacking, and, finally, the use of professional translators or language mediators is not possible 24/7 and involves extra administrative and organisational effort and costs for the healthcare providers. Elements of blended therapy in mother tongue may help clinicians to use time more effectively, increase adherence and maintain changes achieved during face-to-face psychotherapy [10]. Blended care approaches via smartphones may be particularly promising since the majority of refugees use smartphones to access the internet every day [15].

Two recent international implementation studies in Belgium [16] and Canada [17] emphasized hindering factors regarding the implementation process of internet-based applications in inpatient mental healthcare settings. Professionals mentioned high patient turnover in the acute inpatient setting [16], lack of time and a high workload for professionals, technical difficulties and insufficient structural facilities for the use of e-mental health as barriers [17]. Insufficient staff qualification, lack of training and supervision, and other organizational and administrative factors may also hinder the clinical implementation of internet-based applications [18]. The potential of internet-based support in inpatient mental healthcare has therefore not been fully utilized. Guided support by health professionals leading to patient recruitment and stabilizing program adherence as well as professional qualification, training and supervision of practitioners seem to be crucial. The process of adapting and implementing apps for inpatient mental healthcare needs to address clinicians, patients and administrative staff [19]. To date, many clinical practitioners in inpatient settings still lack practical experience with e-mental health care [20]. The gap between the development of effective e-mental health treatments for mental disorders and service provision in routine mental healthcare is considerable. Implementation research is aimed at closing this gap [21].

### Study objectives

The key research question of the present study is whether the newly developed “blended ALMAMAR” app can be successfully implemented in routine clinical inpatient mental healthcare for Arabic- and Farsi-speaking refugees, and whether the app will be accepted and used by patients and professionals. Furthermore, we aim to identify facilitators and barriers to implementation.

## Methods

### Trial design and study setting

This study is part of the I-REACH consortium (**I**nternet-based **Re**fugee ment**a**l **h**ealth **C**are) [https://www.mentalhealth4refugees.de/en/i-reach], which aims to develop and test culturally and contextually adapted internet-based interventions for Arabic- and Farsi-speaking refugees in in- and outpatient settings in Germany. The consortium is funded by a grant from the German Federal Ministry of Education and Research (BMBF) (FKZ: 01EF1806C). This study (subproject 4 of the I-REACH project) focuses on the implementation of blended care using an app in inpatient routine mental healthcare of the psychiatric clinics of the Rhineland Regional Council (Landschaftsverband Rheinland, LVR). The LVR is the largest provider of inpatient mental health care in the Rhineland region. LVR operates nine public psychiatric hospitals, serves 4.5 million inhabitants with a total of 3403 inpatient beds (as of 2020). We chose a routine inpatient setting to ascertain the transferability of the study findings to “real world” mental healthcare. The study is designed as an observational, quasi-experimental, prospective multicenter implementation study. Eight of the nine LVR-clinics participate as recruitment centers in this study and receive case fees for the recruitment and data collection of the patients. The Section Health Services Research at the LVR-Institute for Research and Education is responsible for coordinating the study.

### Inclusion and exclusion criteria

Patients are included based on the following inclusion criteria: (a) adult age (≥ 18 years old), (b) Arabic- or Farsi-speaking refugees, (c) current inpatient mental healthcare in one of the participating LVR-clinics due to depression (codes F32-F34 of the International Classification of Diseases (ICD), tenth revision), anxiety disorder (F40, F41), trauma related disorders (F43) or substance use disorders (F10-F19), and d) written informed consent. Key exclusion criteria are (a) (health) conditions precluding the use of internet-based applications such as blindness, analphabetism, severe intellectual impairment, or (b) involuntary inpatient stay. All refugee patients admitted voluntarily for inpatient mental healthcare for the aforementioned mental disorders are screened for participation.

### Recruitment, participant timeline and informed consent

**Fig. 1:**
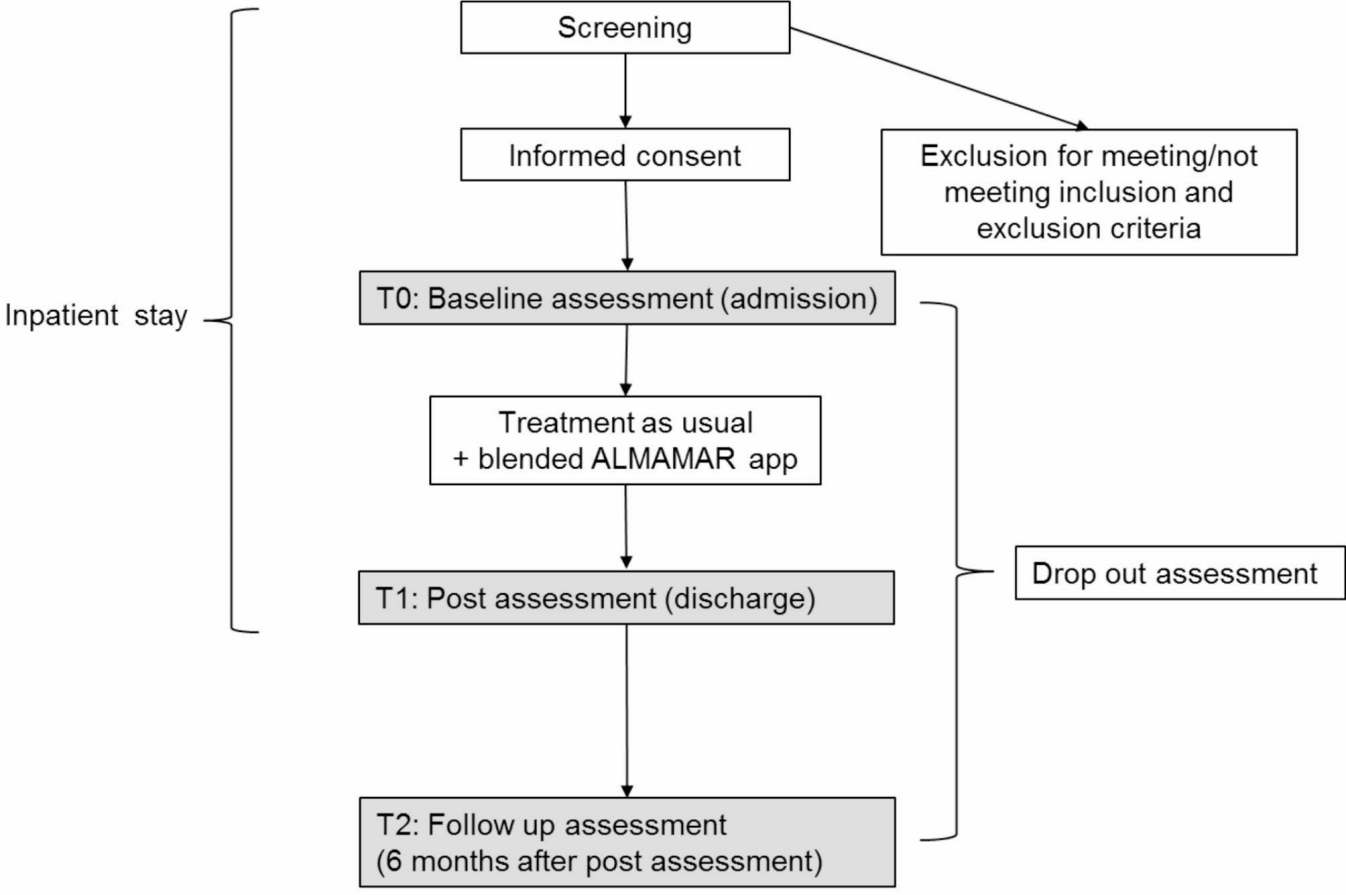
Anticipated patient flow

The recruitment phase started in June 2022 and is planned for 31 months (until December 2024). Physicians and psychologists who work as therapists in the wards and day-clinics of the eight LVR hospitals can participate in the study as treatment providers for the “blended ALMAMAR” app (“therapists”). To familiarize therapists with the app “blended ALMAMAR”, a training course for therapists (two hours) was developed. Having participated successfully in this course, therapists obtain access to the app by an individual account. Therapists recruit patients during inpatient stay (see Fig. 1). Patients can be recruited shortly after admission or at some later point during their inpatient stay. After checking the inclusion and exclusion criteria, therapists hand out study information to suitable participants. A demo version of the “blended ALMAMAR” app can be shown for illustration if needed. Written informed consent sheets are available in Arabic, Farsi and German and interpreters can be consulted. Within the informed consent process, patients will be asked whether they agree to be contacted in case of dropout for participation in an ancillary study of the I-REACH consortium which examines reasons for dropout (“Prediction and prevention of dropout in research, diagnostics, and treatment with refugees”; PrevDrop). In case of consent, a separate informed consent is obtained by the participant for their participation in a qualitative interview. After written informed consent has been obtained, therapists give patients access to the “blended ALMAMAR” app to use it in addition to routine care (i.e., a setting blended of smartphone and personal contacts). All participants of the study fill out standardized questionnaires in Arabic or Farsi at study inclusion (t0: baseline assessment), upon discharge from inpatient treatment (t1: post assessment), and six months after discharge (t2: follow-up assessment). There is no predefined length of participation in the study since the duration of inpatient stay may vary largely between patients. At t0 and t1, questionnaires are delivered via the app. Follow-up assessments (t2) are conducted by telephone or via video call by the therapist (if necessary, with the help of an interpreter). In order to ascertain a high call back rate, therapists note the contact details of patients at discharge and can already fix a date for the planned interview.

### Adjustments of the study design during the course of the project

Some important modifications became necessary in the course of the project:

(1) Initially, when the project was conceptualized in 2017, five of the nine LVR-clinics decided to engage in the blended-care implementation study. As the number of refugees decreased in the following years, we recruited three more clinics. (2) The development of the App (subproject 2 of the I-REACH consortium) was delayed due to the COVID-19 pandemic. Consequently, the implementation study (subproject 4) was delayed. Recruitment of patients started in June 2022 with a delay of 16 months compared to the original plan. (3) Approximately six months after recruitment started, we decided to expand the inclusion criteria by including substance use disorders as an additional diagnostic group. (4) Initially, we had planned a cluster-randomisation with control group design in order to assess potential advantages of the blended care approach compared to treatment as usual (TAU). According to the original study design, half of the recruitment centers should offer TAU and recruit control patients. However, recruitment of a control group proved to be unfeasible in this trial: After six months of futile recruitment efforts, we decided to forgo the control group and focus on the main goal of the planned study, which had been the study of the implementation. Thereafter, the four control group centers were converted into intervention centers. The funding partner, ethics committee and Data Safety Monitoring Board (DSMB) approved this revision of the study concept.

### Intervention

Patients are offered the use of the “blended ALMAMAR” app during their inpatient stay in addition to routine care. The app (named ALMAMAR, Arabic for “path”) is based on CETA (Common Elements Treatment Approach) [19], a transdiagnostic therapeutic intervention that has already been evaluated in different settings and groups [e.g. 22–24]. CETA was adapted for the target group within subproject 2 of the I-REACH consortium [25]. Thereafter, the intervention was converted into a digital format (ALMAMAR) and thereafter, it was adapted to the inpatient setting (“blended ALMAMAR”). The adaptation process from ALMAMAR to “blended ALMAMAR” was performed by a multi-professional team from subprojects 2 and 4, which combined expertise in psychiatry, psychology and computer science. Each part (= “module”) of the app was checked for suitability for the inpatient context regarding (a) fitting to needs of inpatient care, (b) content, (c) complexity, (d) interactive character of the exercise, (e) duration, and e) phrasing.

The “blended ALMAMAR” app is able to stand alone (stand-alone self-management). Hence, patients may work with the app independently from the direct feedback of their therapists. The “blended ALMAMAR” version of the app was developed as an information and/or psychoeducation tool, and as an aid for the communication between therapist and patients. By reading information texts offered digitally in their native language and completing worksheets, patients can identify stress factors and resources, and they gain information about their mental illness. Therapists have the opportunity to view the digitally completed worksheets of their patients and receive valuable information that they can use in face-to-face-therapy, if necessary with the support of an interpreter. As automatic translation was not integrated into the app and Arabic and Farsi speaking practitioners or interpreters are not available 24/7 in routine inpatient mental health care, we had to ascertain that patients do not use the app for messages to their therapists, e.g. about suicidal thoughts. Accordingly, we did not allow free text entries within the “blended ALMAMAR” app. Instead, patients choose between predefined answer categories.

### Adaptations from the original CETA

Altogether, the “blended ALMAMAR” app for the inpatient setting comprises seven modules: *introduction, cognition, difficult memories, activities, substance use, relaxation techniques*, and *closing* (see Table 1 for details and adaptations from the original CETA). Two modules from the original CETA manual requiring direct interaction or free text entries (e.g., writing a letter to a friend) (modules “safety” and “exposure”) were not transferred into the blended ALMAMAR app. Both modules are performed in a face-to-face setting during inpatient treatment, if indicated. Additionally, some more complex and interactive cognitive exercises from the CETA manual were not adapted for the “blended ALMAMAR” app. In contrast to the original CETA program, there is no predefined order (‘flow’) or frequency of module usage by patients. Depending on the individual needs of their patient, therapists may choose between a standardized flow of modules and exercises for a specific mental disorder (e.g., depression flow, addiction flow etc.) and an individual flow consisting of an introductory module and other modules and exercises that can be added from a list (‘module pool’). Adaptations of the chosen flow are possible throughout the treatment. The duration of usage of the app is not predefined. The “dosage” prescribed (= number of modules and exercises selected by the therapist for the patient) depends on the clinical judgment of the therapist, there is no maximal or minimal dosage needed. The therapists are able to monitor the progress in the app usage of their patients and they can see all ratings of their patients in the German language (due to the predefined answers, the app can also show the Arabic/Farsi answers in German). Patients are able to continue working with the app during their inpatient stay, independent of face-to-face feedback from the therapist. The app can only be used during inpatient stay and is deactivated at discharge by the therapist. At discharge or in case of dropout, therapists have to manually select the module ‘closing’ in the app which contains a summary of all modules that the patient worked on as well as concluding words and all patient questionnaires for the post assessment.

**Table 1:** Overview of the modules and content of the blended ALMAMAR app

No	Module	Content	Adaptation from original CETA
1	Introduction and explanation “blended ALMAMAR” app, overview of the planned modules	Overview of the goal and benefits of the “blended ALMAMAR” app as well as the modules. In addition, possible concerns and barriers to treatment are presented using pre-defined categories. Patients can select categories that apply to them by clicking on them. The module contains the questionnaires Baseline, which have to be filled out by the patients.	Adaptation of “encouraging participation” by using predefined categories; “introduction” was adapted to context of inpatient setting
2	Cognition: explaining the relationship between thoughts, feelings and behavior.	In this module, knowledge about the connections between thoughts, feelings and behavior is conveyed using example situations. Feelings and behaviors resulting from “helpful” and “unhelpful” thoughts will be reflected. The users can choose from predefined lists of example situations, corresponding thoughts and behaviors that they are familiar with or would be willing to try out.	Some of the more complex/interactive cognitive tasks were omitted (“thinking in a different way”; “family/friend role play”; “letter to a friend”; “responsibility pie”)
3	Difficult memories (lifeline)	Here, knowledge is imparted about the connections between important life events and feelings and behavior. It is possible to create a “lifeline” with significant (positive and negative) life events. Using predefined lists, life events can be selected and transferred to a timeline. The life events can be evaluated in terms of their significance.	“Lifeline” was used with predefined categories. We chose to add positive events to the exercise.
4	Behavioural activation (part 1 and part 2)	Part 1: Here, knowledge about the importance of stress-reducing activities is provided and a list of pleasant activities can be created based on predefined categories. For each entry on the list, lists of obstacles to this activity and possible solutions (again, selection from predefined categories) can also be created. The activity can then be entered into the calendar. Scheduled activities then appear in the calendar. Part 2 of this module includes an assessment of the completed activity selected in Part 1. Patients then have the opportunity to plan and complete the activity from the previous week or a new activity.	Only predefined categories of activities are used.
5	Substance use (part 1 and 2)	This module is about, teaching the connections between use (alcohol consumption/drug use/gambling addiction) and mental health impairments, feelings that arise before, during, and after substance use/gambling as well as positive and negative effects of substance use/gambling addiction. The module also aims to change attitudes/behaviors regarding substance use/gambling addiction. Patients have the opportunity to select a strategy/technique/interactive exercise (letting the urge go away, finding new activities, etc.). Patients can also repeat exercises and do more exercises (part 2 of the module).	The “addiction information sheet” is not used, all other exercises are carried out with predefined categories
6	Relaxation techniques (part 1 and part 2)	In this module, knowledge about different relaxation techniques is imparted. At the beginning, it is possible to choose from a list of relaxation techniques which techniques are already familiar. Each relaxation technique is rated on how helpful it is/was. Two relaxation techniques (Breathing and Grounding) are also integrated as audio files and can be performed afterwards and scheduled in the calendar. The exercise then appears in the calendar.	The relaxation techniques are presented through audio files.
7	Finishing steps	Summary of the completed modules. The module also contains the questionnaires that patients are asked to complete at the time of discharge. In addition, patients rate whether they have learned anything by using “blended ALMAMAR” during inpatient treatment.	“Talking about next steps” had to be omitted (patients have no access to the app and no contact to the therapist after discharge).

### Assessments and outcomes

The main hypothesis of the study is that the “blended ALMAMAR” app can be successfully implemented in inpatient psychiatric-psychotherapeutic care of refugees, and that it is accepted and used by therapists and patients.

The *primary endpoint* will be the use of the “blended ALMAMAR” app during inpatient stay, measured by:

- recruitment rate (proportion of eligible patients who signed a consent form to participate);
- app usage rate (proportion of recruited patients who use the app at least once);
- average number of modules completed in the app;
- average duration and frequency of use of the app.

*Secondary endpoints* are clinical assessments, as well as acceptability and usability of the intervention. These will be assessed using questionnaire data from patients and therapists at each of the three time points (baseline assessment at study entry t0, post assessment at discharge t1, and follow-up six months after discharge t2; see Table 2 for an overview of all assessments).

**Table 2:** Overview of the planned assessments

	Instrument	Who fills in?	Timepoint of measurement			Estimated duration (minutes)
No.			T0: Baseline (Admission)	T1: Post (Discharge)	T2: Follow-up (six months after discharge)	
1	Sociodemographic data	Patient	x			10
2	User rating of the app (CSQ-8) [30]	Patient		x	x	5
3	Post Migration Living Difficulties Checklist (PMLD) [26]	Patient	x			10
4	Expectation of therapy	Patient	x			5
5	EQ-5D-5 L (Health-related quality of life) [27]	Patient	x	x	x	5
6	*Health care utilization [selected questions from FIMA* [28] *and FIMPsy* [29]:					
	Number and specialisation of outpatient physician contacts in the last six months before inpatient admission (or baseline) and in the six months after discharge	Patient	x		x	3
	Number of inpatient stays and treatment days in the last six months before inpatient admission (or baseline) and in the six months after discharge	Patient	x		x	3
	Diagnoses, medications	Patient	x		x	3
	Number of visits to emergency departments in the last six months before hospitalization (or baseline) and in the six months after discharge	Patient	x		x	1
	Current employment [yes/no]	Patient	x		x	3
7	Main diagnosis of mental disorder and secondary diagnosis(es)	Therapist	x	x		1
8	Number of inpatient psychiatric treatment days and stays in the last six and twelve months, respectively	Therapist	x	x	x	3
9	Symptom severity (CGI) [31] and functional ability (GAF) [32]	Therapist	x	x		5
10	Regular insurance status [yes/no]	Therapist	x			1
11	Assessment of therapy success [rating 1–5]	Therapist		x		1

At baseline, patients fill out questionnaires on post-migration stressors (27-items post-migration living difficulties checklist (PMLD); [26]), expectations for the current therapy (3 items; self-developed questionnaire), and health-related quality of life (EQ-5D-5 L; [27]). Additionally, we assess costs of health care utilization by selected questions from the FIMA [28] and FIMPsy [29] questionnaires by asking about the number of physician contacts and inpatient stays within the last six months before admission/baseline and about the duration of these inpatient stays, past diagnoses, medication and current employment status. Asylum-related factors (e.g. length of stay, asylum status) are assessed at baseline. At discharge, patients fill in a user rating of the app (adapted version of the CSQ-8; [30]) as well as the health-related quality of life (EQ-5D-5 L; [27]).

Therapists will indicate the current main diagnosis of mental disorders as well as secondary diagnoses. In addition, they will perform clinical ratings of symptom severity (CGI; [31]) and functional ability (GAF; [32]) at each assessment. They will indicate the number of inpatient psychiatric treatment days (at post-assessment) and perform a rating of overall therapy success at discharge (rating 1–5). In case of dropout, therapists document reasons. In a separate assessment, therapists will be asked about their attitudes/expectations and experiences with digital interventions before working with the app (pre-assessment) and after approximately 12–24 months of experience with the app (post-assessment). For this assessment, we created short questionnaires that are given to all therapists after an initial app training session.

### Implementation process

We use the RE-AIM model (reach, efficacy, adoption, implementation, maintenance) as a theoretical framework for the implementation process. This model was originally designed for evaluating interventions to improve general health [33]. It was later adapted for evaluating informatics-based applications in clinical settings [34], and has already been successfully applied [35]. Bakken and co-authors define five dimensions: (1) *Reach* describes the absolute number and proportion of people receiving the intervention and its representativeness of the population; (2) *Efficacy* measures the degree of impact of the intervention in terms of quality of care (for processes and outcomes) and observes unanticipated positive or negative effects of the intervention; (3) *Adoption* refers to the absolute number and proportion of individuals who forgave the intervention and its representativeness of the population; (4) *Implementation* describes, at the setting level, the adherence to the intervention by those who prescribe the intervention, i.e., the consistency of administering the intervention as scheduled, as well as associated time and cost; additionally, at the individual level, the behavior of patients in terms of using the intervention strategies; (5) *Maintenance* includes, at the setting level, the degree to which the intervention is adopted in routine care and, at the individual level, the long-term effects of a program on specific outcomes at six or more months after the last intervention contact. When planning the study, these dimensions of the theoretical model were taken into account. An evaluation of the implementation process according to the dimensions is planned at the end of the project.

### Sample size

This is an implementation study investigating the feasibility and perceived benefits of a nonmedication intervention under routine clinical conditions. The focus is on feasibility and acceptability of the implementation of an app in routine mental health care (blended care) for refugees, not on clinical effectiveness. Across all eight recruitment centers (LVR-clinics), we aim to recruit 100 patients for this study and assume a dropout rate of 20%.

### Ethical aspects

The study protocol as an implementation study and its amendments were approved by the responsible ethics committee (Medical Association of North Rhine [Ärztekammer Nordrhein]; Germany; EK2021113 on July 21, 2021; amendments 6000251823 and 6000255968).

### Data management and data access

All data protection regulations of the German Federal and State Data Protection Acts as well as the General Data Protection Regulation (Datenschutzgrundverordnung, DSGVO) are taken into account within the framework of a data protection concept. A data safety plan was approved by the ethics committee. The data protection requirements have also been agreed upon by the Rhineland State Council (LVR) data protection officer. All data will be directly collected within the “blended ALMAMAR” app. The LVR-Institute for Research and Education (study coordination center) is responsible for quality control and data management. When exporting data from the app to external partners, no personally identifying data (usernames or contact data) are exported. Instead, each data set receives a randomly generated ID and is thus pseudonymized. Anonymous data export (excluding personal data) is currently only planned for partners within the I-REACH consortium and the ancillary project PrevDrop.

### Confidentiality

Patients and therapists will register in the “blended ALMAMAR” app with their E-mail address and/or phone number (for patients optionally, necessary for password reset). All data are stored on a secure platform, and no personal data will be exported from the app. After the trial, data will be stored for ten years. Patients will receive extensive information on data assessment, transfer, storage and access policy within the informed consent procedure.

### Statistical methods for primary and secondary outcomes

Descriptive statistics will be performed for all quantitative variables at t0, t1 and t2. For all categorical variables, numerical and frequency data will be calculated. The main outcome variables (recruitment rate, app usage rate, average number of modules completed in the app, average duration and frequency of use of the app) are descriptive data that can be analysed by recruitment documentation of the clinics as well as log-in data from the app. Regarding secondary outcome variables, we will perform pre-post analyses regarding CGI and GAF as well as health-related quality of life using Wilcoxon test. Intention-to-treat (ITT) and per protocol analysis will be conducted to avoid selective reporting and biases in the analyses. Missing values will be imputed by the appropriate statistical methods depending on the type of missing. FIMA und FIMPsy will be used to measure healthcare utilization which will be multiplied with unit costs to calculate costs. Using these costs, a budget impact analysis will be conducted to analyse the cost impact of introducing “blended ALMAMAR” into routine care.

Subgroup analyses will be performed (e.g. regarding diagnoses and provider location), and predictors and mediators (e.g. PMLD or health-related quality of life scores) will be included as covariates. SPSS software will be used for data analyses and two-sided p-values of p < .05 will be accepted as statistically significant.

### Oversight and monitoring

Access to the app is restricted to therapists (physician and psychologists) who were trained in its use. Detailed information material (“blended-ALMAMAR” manual) is provided by the study coordination center. Additionally, every clinic has appointed one responsible local coordinator. The local coordinators were trained ito be able to overview and supervise recruitment, data collection and adherence to the study protocol at the level of the single recruitment center. There is no independent Trial Steering Committee. The research team in the study coordination center reviews recruitment rates, data collection and data quality for all clinics on a regular basis and meets with all local coordinators of the clinics on a monthly basis to discuss key milestones as well as recruitment strategies. Regular monitoring visits will take place in every recruitment center, and a standardized procedure has therefore been developed. All data is coded directly into the database and all action are logged. Significant amendments to the original study protocol were provided to and approved by the responsible ethics committee.

### Patient safety and management of adverse events

Participation in the study is voluntary and consent to participate can be withdrawn without giving reasons and without any disadvantages for patients. After reviewing the inclusion and exclusion criteria, therapists decide whether patients will be given access to the “blended ALMAMAR” app. All online modules are carried out in an interlinked treatment model (blended care) only after the therapist responsible for the case has activated the app for the respective patient. The manufacturer of the app (Freie Universität Berlin) checked both the technical and the content components of the app (e.g. translations) as part of its quality assurance. Although the overall risk of side effects or adverse events in the context of this study is low, the use of the app may be associated with some risk of worsening psychological well-being [36]. Patients may be psychologically distressed by the app content or by the completion of questionnaires. Since the patients are undergoing inpatient psychiatric-psychotherapeutic treatment, the responsible therapist can take action promptly if the health condition deteriorates or other problems occur. The risk of patients participating in the study is therefore considered to be low. In addition, an independent Data Safety and Monitoring Board (DSMB) was established. The DSMB is independent of all investigators and funders, and no member of the DSMB is directly involved in the conduct of the study.

### Dissemination plans

The study results will be published in an open access peer-reviewed journal and they will be presented at national and international scientific conferences. We will use our website to disseminate project results. After termination of the study, we aim to make the app available for open-access use for inpatient treatment. For inpatient services, we will prepare a comprehensive implementation manual that addresses all practical implementation issues and offers solutions to overcome potential barriers of routine clinical implementation.

## Discussion

Internet-based applications have the potential to improve healthcare for vulnerable groups, but the implementation of new approaches is often challenging. The aim of this study is to investigate blended care by using the “blended ALMAMAR” app during inpatient routine mental health care of refugees in Germany. The newly developed app may help to close communication gaps for the hard-to-reach and vulnerable patient group of refugees in inpatient treatment. “Blended ALMAMAR” will be the first e mental health intervention to address severely mentally ill refugees in an inpatient setting. The “blended ALMAMAR” app has the potential to enhance mental health treatment for refugees by providing information in their mother tongue and lowering the need for support by interpreters. We aim to explore whether the app will be used and accepted by therapists and patients in a clinical routine care setting in Germany. Hence, the main research questions of this implementation study focus on the usage and acceptance of the app by therapists and patients as well as the implementation process itself.

At the level of the patients with refugee backgrounds included in this study, we do not restrict participation to a certain length of stay in Germany or to an (approved) asylum status. Asylum-related factors will nevertheless be assessed and taken into account in the analysis of the results of the study. This wider inclusion criterion will make the group of participants more heterogenous, but it goes along with the aim of the study to close a gap for refugees in inpatient mental health care treatment and it will also contribute to the external validity of the study.

### Trial status

Patient recruitment started in June 2022 and is expected to be completed by December 2024.

## Data Availability

Not applicable.

## Abbreviations

CETA: Common Elements Treatment Approach
CGI: Clinical Global Impression Scale
DSGVO: Datenschutzgrundverordnung (General Data Protection Regulation)
DSMB: Data Safety and Monitoring Board
EQ-5D-5L: European Quality of Life 5 Dimensions 5 Level Fragebogen zur Inanspruchnahme von medizinischen und nicht-medizinischen Versorgungsleistungen im Alter (Questionnaire on the Utilization of Medical and Non-Medical Services for the Elderly)
FIMPsy: Fragebogen zur Inanspruchnahme von medizinischen und nicht-medizinischen Versorgungsleistungen bei psychischen Störungen (Questionnaire on the Utilization of Medical and Non-Medical Services for Mental Disorders)
GAF: Global Functional Ability Scale
I-REACH: Internet-based Refugee mental health Care
ITT: intention-to-treat
LVR: Landschaftsverband Rheinland
PrevDrop: Prediction and prevention of dropout in research, diagnostics, and treatment with refugees
PMLD: Post-migration living difficulties checklist
PTSD: post-traumatic stress disorder
RCT: randomized controlled trial
